# Synthesis, Characterization, and Handling of Eu^II^-Containing Complexes for Molecular Imaging Applications

**DOI:** 10.3389/fchem.2018.00065

**Published:** 2018-03-19

**Authors:** Lina A. Basal, Matthew J. Allen

**Affiliations:** Department of Chemistry, Wayne State University, Detroit, MI, United States

**Keywords:** chelating ligands, contrast agents, coordination chemistry, divalent europium, lanthanides, molecular imaging probes

## Abstract

Considerable research effort has focused on the *in vivo* use of responsive imaging probes that change imaging properties upon reacting with oxygen because hypoxia is relevant to diagnosing, treating, and monitoring diseases. One promising class of compounds for oxygen-responsive imaging is Eu^II^-containing complexes because the Eu^II/III^ redox couple enables imaging with multiple modalities including magnetic resonance and photoacoustic imaging. The use of Eu^II^ requires care in handling to avoid unintended oxidation during synthesis and characterization. This review describes recent advances in the field of imaging agents based on discrete Eu^II^-containing complexes with specific focus on the synthesis, characterization, and handling of aqueous Eu^II^-containing complexes.

## Introduction

**Figure 1:**
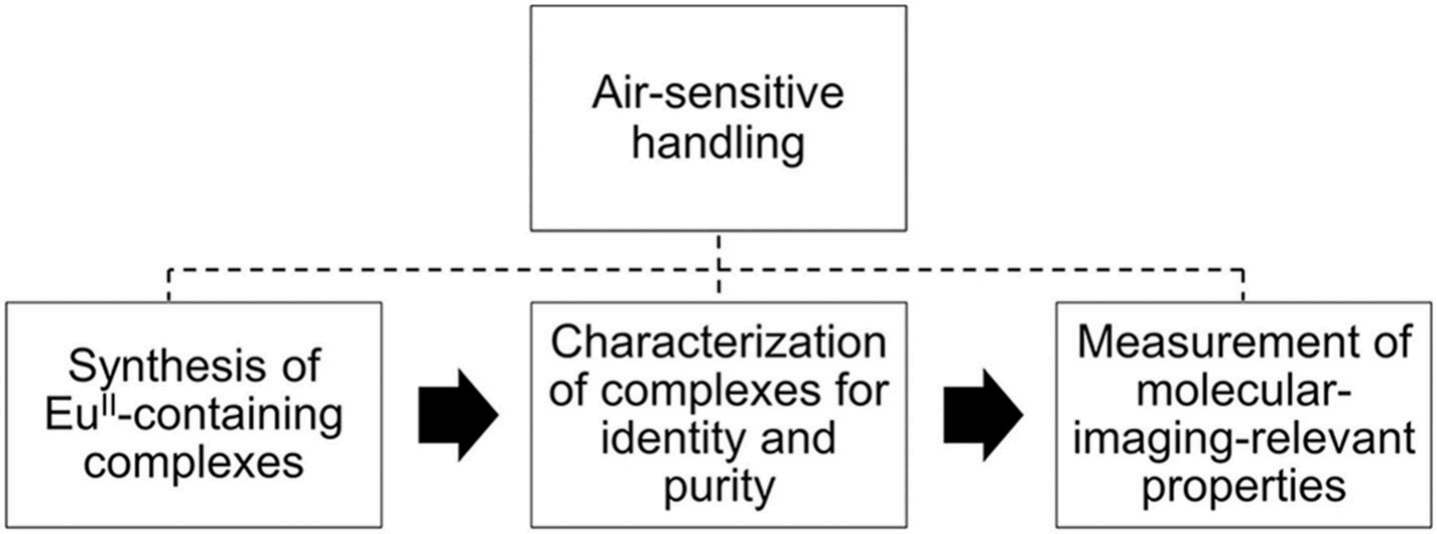
Pathway for Eu^II^-containing complexes that have applications in molecular imaging.

Divalent europium is a paramagnetic ion with photophysical and electrochemical properties that can be modulated with coordination chemistry (Gansow et al., 1977; Yee et al., 1980, 1983; Sabbatini et al., 1982, 1984; Antonio and Soderholm, 1996; Jiang et al., 1998; Shipley et al., 1999; Burai et al., 2000; Soderholm et al., 2002; Botta et al., 2003; Christoffers and Starynowicz, 2008; Gamage et al., 2010; Pan et al., 2011; Garcia and Allen, 2012a,b; Gál et al., 2013; Kelly et al., 2015; Kuda-Wedagedara et al., 2015; Regueiro-Figueroa et al., 2015; Allen, 2016; Ekanger et al., 2016a, 2017; Jin et al., 2016; Vanek et al., 2016; Basal et al., 2017a,b; Burnett et al., 2017; Kawasaki et al., 2017; Corbin et al., 2018; Jenks et al., 2018). Because of these tunable properties, divalent europium complexes have potential use as molecular-imaging probes that sense O_2_, which is important because O_2_ imbalances are often correlated with disease (Shweiki et al., 1992; Barnham et al., 2004; Mattson, 2004; Lin and Beal, 2006; Park et al., 2008; Facciabene et al., 2011). One promising technique to investigate oxygenation is contrast-enhanced magnetic resonance imaging (MRI) with Eu^II^-containing complexes. Divalent europium was proposed as a *p*O_2_ sensor for MRI (Burai et al., 2000) because divalent europium acts as a *T*_1_-shortening contrast agent for MRI until it is oxidized to Eu^III^, which shows no measurable *T*_1_-shortening ability at imaging-relevant concentrations and field strengths (≤6 mM) (Ekanger et al., 2016a; Basal et al., 2017b). Recently, the first example of *in vivo* imaging using a Eu^II^-containing contrast agent was reported (Ekanger et al., 2015), and other *in vivo* examples followed (Ekanger et al., 2016b; Funk et al., 2016; Basal et al., 2017a). The recent advancement of Eu^II^-containing complexes as O_2_ sensors for molecular imaging and the unique experimental challenges of characterizing and handling air-sensitive Eu^II^-containing complexes inspired this review. Unlike Gd^III^, which is a commonly studied ion for MRI, Eu^II^ is air-sensitive and requires careful handling techniques because Eu^II^ oxidizes to Eu^III^ when exposed to O_2_. Many researchers who might be interested in Eu^II^ likely have experience with Gd^III^. However, because of the crucial differences in preparation and handling between Eu^II^ and Gd^III^, this review focuses on the synthesis, handling, and characterization of identity and purity of Eu^II^-containing complexes relevant to molecular imaging (Figure 1). For measurements of molecular-imaging-relevant properties for MRI, readers are referred elsewhere (Caravan et al., 1999; Burai et al., 2000, 2002; Tóth et al., 2001; Botta et al., 2003; Garcia et al., 2011, 2012; Garcia and Allen, 2012b; Ekanger et al., 2014, 2015, 2016a,b; Basal et al., 2017a,b; Lenora et al., 2017; Pierre et al., 2018). Because this review is focused on techniques for handling and characterizing discrete, air-sensitive Eu^II^-containing complexes for molecular imaging, we do not describe other divalent lanthanide complexes, nanoparticles, imaging probes, or nonaqueous Eu^II^-containing complexes. Readers are referred elsewhere for details of those subjects (Evans, 2000; Dorenbos, 2003; Bochkarev, 2004; Bottrill et al., 2006; le Masne de Chermont et al., 2007; Kuda-Wedagedara and Allen, 2014; Pierre et al., 2014; Ekanger and Allen, 2015; Angelovski, 2016; Edelmann, 2016; Wang et al., 2017).

## Synthesis of Eu^II^-containing complexes

**Figure 2:**
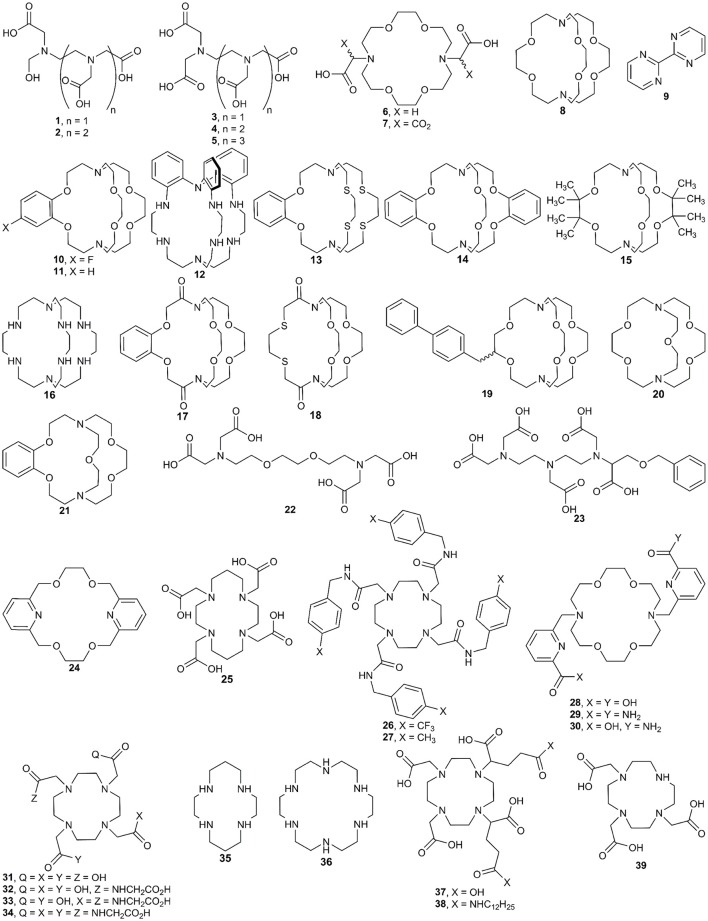
Ligands used with Eu^II^ and the techniques described in the synthesis section of this manuscript.

**Figure 3:**
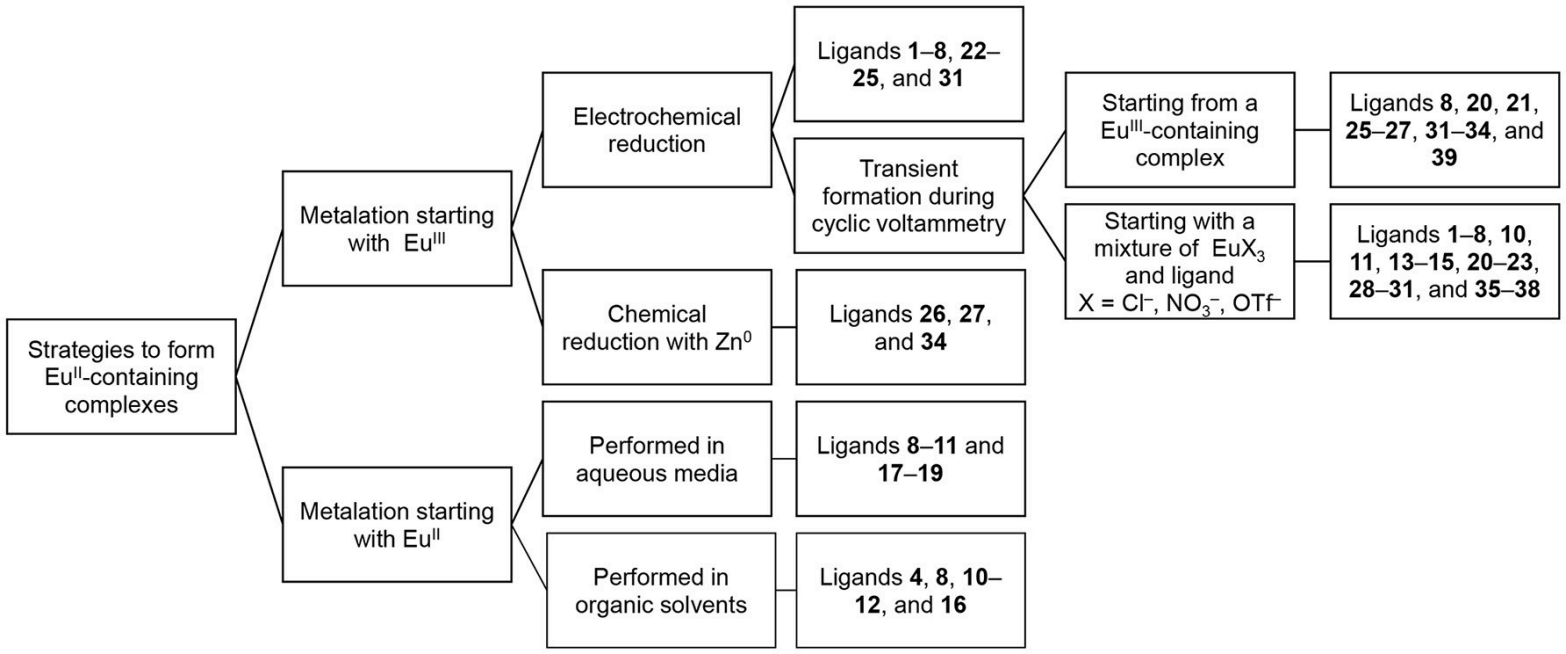
Reported strategies to form the Eu^II^-containing complexes included in this manuscript.

In this section, we describe procedures for the preparation of Eu^II^-containing complexes using the ligands depicted in Figure 2. These procedures are divided into two general strategies (Figure 3): (1) chemical or electrochemical reduction of Eu^III^-containing complexes or mixtures of Eu^III^ salts and ligands and (2) metalation of ligands with Eu^II^ salts. Depending on the route used to generate Eu^II^-containing complexes, different techniques are appropriate to evaluate the identity and purity of the resulting complexes. These characterization techniques and strategies for effectively handling solutions of Eu^II^-containing complexes for analyses are described in the handling section of this article.

### Reduction of Eu^III^ to produce Eu^II^-containing complexes

In the process of forming Eu^II^-containing complexes, Eu^III^ is often reduced using electrochemical or chemical methods. Transient formation of detectable amounts of Eu^II^-containing complexes can be achieved using cyclic voltammetry, and isolable Eu^II^-containing complexes can be obtained through electrochemical or chemical reduction of either Eu^III^-containing complexes or mixtures of Eu^III^ with ligands. The most favorable route to form a Eu^II^-containing complex should be determined based upon the ligand and the type of analysis that is needed.

#### Transient formation of Eu^II^-containing complexes from cyclic voltammetry

**Figure 4:**
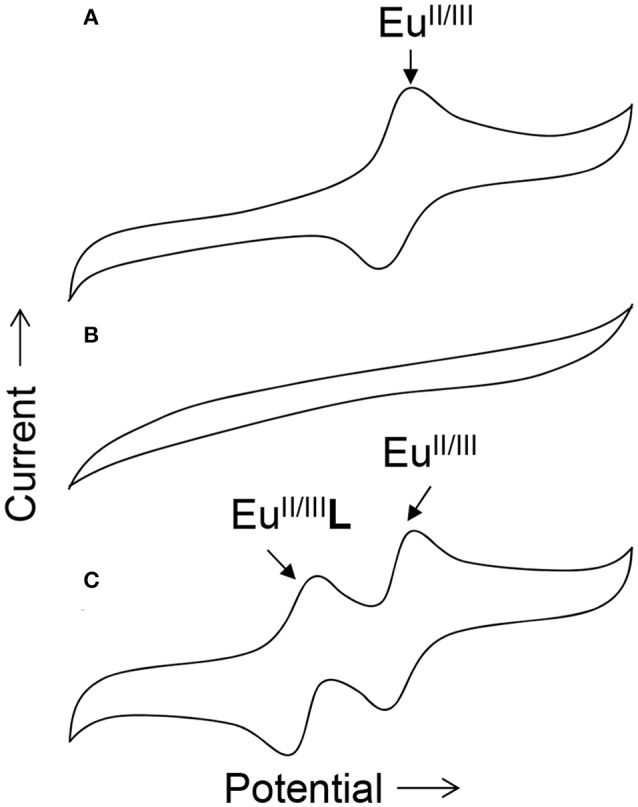
Cartoon cyclic voltammograms of **(A)** a solution of ${\text{Eu}}_{\text{aqua}}^{\text{II}/\text{III}}$ in water, **(B)** a solution of ligand **L** in water, and **(C)** a solution of both ${\text{Eu}}_{\text{aqua}}^{\text{II}/\text{III}}$ and **L** in water. The new peak in **(C)** relative to **(A)** is indicative of the *in-situ* formation of the Eu-containing complex Eu**L**.

To obtain information about the reduction and oxidation potentials of Eu^II^-containing complexes, several research groups have formed Eu^II^-containing complexes transiently using cyclic voltammetry. A description of air-free electrochemical techniques used for cyclic voltammetry or bulk electrolysis to form Eu^II^-containing complexes is described in the handling section of this review. When reducing Eu^III^ to form Eu^II^, two routes are commonly taken: the corresponding Eu^III^-containing complexes are synthesized and purified before electrolysis, such as in the case of Eu-containing complexes of **8**, **20**, **21**, **25**, **26**, **27**, **31–34**, and **39** (Gansow et al., 1977; Yee et al., 1980, 1983; Burai et al., 2003; Vanek et al., 2016; Basal et al., 2017a; Burnett et al., 2017). Alternatively, Eu^III^ salts—such as Eu(OTf)_3_, EuCl_3_, or Eu(NO_3_)_3_–are dissolved in the presence of ligands, enabling the formation of complexes upon electrolysis of Eu^III^ to Eu^II^, such in the case of Eu-containing complexes of **1–8**, **10**, **11**, **13–15**, **20–23**, **28–30**, and **35–38** (Yee et al., 1980, 1983; Sabbatini et al., 1984; Burai et al., 2002, 2003; Botta et al., 2003; Gamage et al., 2010; Gál et al., 2013; Regueiro-Figueroa et al., 2015). In these experiments, cyclic voltammetry peaks that are different than the peaks for ${\text{Eu}}_{\text{aqua}}^{\text{II}/\text{III}}$ or the ligand (if the ligand is redox active in the potentials spanned by the voltammogram) are attributed to the formation of Eu^II/III^-containing complexes (Figure 4).

For the selection of an appropriate route, consideration of ligand structure and solubility is necessary. For example, ligands that readily form complexes with Eu^II^ upon reduction from Eu^III^, like cryptands **8**, **20**, and **21**, produce the same electrochemical profiles whether starting with a mixture of Eu^III^ and ligand or a Eu^II^-containing complex (Yee et al., 1980, 1983). When solubility differences exist between ligands and their corresponding complexes, such as if the ligand is not soluble but the complex is, then one must ensure complexation prior to CV analysis. If the complex is insoluble in aqueous media, then organic solvents can be employed with the caveat that the measured standard electrode potential might not be reflect the potential under aqueous conditions.

#### Producing Eu^II^-containing complexes via electrochemical reduction of Eu^III^

Beyond the transient reduction of Eu^III^ on the surface of electrodes during cyclic voltammetry, Eu^III^ can be reduced on an isolable scale electrochemically using bulk electrolysis in oxygen-free solvent under an atmosphere of inert gas. Reduction via bulk electrolysis involves holding a sufficiently negative potential to reduce Eu^III^ to Eu^II^. The electrochemical potential used to reduce a Eu^III^-containing complex is often 0.1–0.5 V more negative than the *E*_1/2_ of the target complex (Burai et al., 2002, 2003; Botta et al., 2003); however, the reduction potential of the ligand functional groups should be considered before selecting this technique to avoid the possibility of reducing redox-active ligands. Bulk electrolysis was used to obtain Eu^II^-containing complexes of **1**–**8**, **22**–**25**, and **31** (Sabbatini et al., 1982, 1984; Burai et al., 2000, 2002; Christoffers and Starynowicz, 2008). In these studies, solutions containing both Eu^III^ salts and ligands were held at the appropriate potential, typically in a two-compartment glass cell with a fritted glass separator with sparging of inert gas (Bard and Faulkner, 2000). The resulting Eu^II^-containing complexes can be used for further analysis of molecular-imaging-relevant properties, including UV–visible and luminescence spectroscopy, relaxivity measurements, ^17^O-NMR spectroscopy, and NMRD measurements (Sabbatini et al., 1984; Burai et al., 2000, 2002; Christoffers and Starynowicz, 2008). Bulk electrolysis of a solution of metal and ligand can provide enough material to obtain crystals for X-ray diffraction: for example, bulk electrolysis of Eu^III^ to Eu^II^ in the presence of ligand **24** followed by slow evaporation or cooling under inert atmosphere resulted in crystals of Eu^II^**24** (Christoffers and Starynowicz, 2008). Bulk electrolysis to produce isolable Eu^II^-containing complexes is appropriate when the potential needed to reduce a Eu^III^-containing complex to a Eu^II^-containing complex does not overlap with the redox-activity of the ligand, when the desired Eu^II^ salt is unavailable, or when the standard potential or pH of the complex in solution is incompatible with chemical reductants.

### Chemical reduction of Eu^III^-containing complexes to form Eu^II^-containing complexes

In addition to bulk electrolysis, chemical reductants are used to generate Eu^II^-containing complexes. Depending on the standard potential of the Eu^III^-containing complex to be reduced, different reducing agents will be appropriate. For example, the reduction potential of Zn (Zn^II^ + 2e^−^ → Zn^0^) is −0.960 V vs Ag/AgCl (saturated KCl) (Bard and Faulkner, 2000); therefore, complexes that have standard electrode potentials more positive than −0.960 V vs Ag/AgCl should, thermodynamically, be reduced by Zn^0^. Eu^III^-containing complexes were reduced using Zn^0^ to form Eu^II^**26**, Eu^II^**27**, and Eu^II^**34** (Ekanger et al., 2016a; Basal et al., 2017a,b). In these studies, the Eu^III^-containing complexes were dissolved in water in the presence of zinc metal dust, and the pH was adjusted between 4 and 6.5 to expose Zn^0^, resulting in the reduction of Eu^III^ to Eu^II^. To date, only amalgamated Zn and Zn^0^ have been used to chemically reduce Eu^III^-containing complexes to Eu^II^-containing complexes in water (McCoy, 1935; Ekanger et al., 2016a; Basal et al., 2017a,b). However, other chemical reductants, which have been used to reduce other Ln^III^ ions to Ln^II^ ions (Teprovich et al., 2008; MacDonald et al., 2013; Fieser et al., 2015), could be used if the low pH required for the use of zinc metal is undesirable or if the standard electrode potential of the Eu-containing complex is more negative than that of Zn^0^.

### Complex formation by direct mixing of Eu^II^ salts and ligands

Another technique to synthesize Eu^II^-containing complexes is mixing Eu^II^ halide salts with ligands. Eu^II^ chloride, bromide, and iodide salts are available commercially. When mixing Eu^II^ and ligands, often a slight excess of a Eu^II^ halide salt (1.1–1.2 equivalents) is mixed with a water-soluble ligand (1 equivalent) in water. Complexes tend to be easier to purify from an excess of Eu^II^ relative to an excess of ligand: the addition of phosphate buffer precipitates excess Eu^II^ from solution as phosphate salts that can be removed with a small (0.2 micrometer) hydrophilic filter to yield a buffered solution of Eu^II^-containing complex (Garcia and Allen, 2012b). This technique was used to synthesize Eu^II^-containing complexes of **8**–**11** and **17**–**19** (Zucchi et al., 2010; Garcia and Allen, 2012b; Garcia et al., 2012; Ekanger et al., 2014, 2015, 2016b, 2017; Lenora et al., 2017).

When a ligand is not water-soluble but the resulting complex is, aqueous solutions of Eu^II^-containing complexes can be prepared by mixing Eu^II^ salts with ligands in an organic solvent and then separating the resulting complex from the organic solvent. Purification by precipitation or crystallization results in solids that are soluble in water for imaging. For an example of purification by precipitation, Eu^II^**16** was synthesized in tetrahydrofuran: EuI_2_ and **16** are soluble in tetrahydrofuran, but Eu^II^**16** is not, enabling isolation of Eu^II^**16** by precipitation (Kuda-Wedagedara et al., 2015). For an example of purification by crystallization, crystals were grown of cryptates Eu^II^**4**, Eu^II^**8**, Eu^II^**10**, Eu^II^**11**, Eu^II^**12**, and Eu^II^**16** from slow evaporation of a mixture of ligand and Eu^II^ halide in acetone, methanol, or methanol/tetrahydrofuran (Burai et al., 2000; Ekanger et al., 2015; Kuda-Wedagedara et al., 2015; Jin et al., 2016; Lenora et al., 2017).

## Characterization for identity and purity in aqueous media

**Figure 5:**
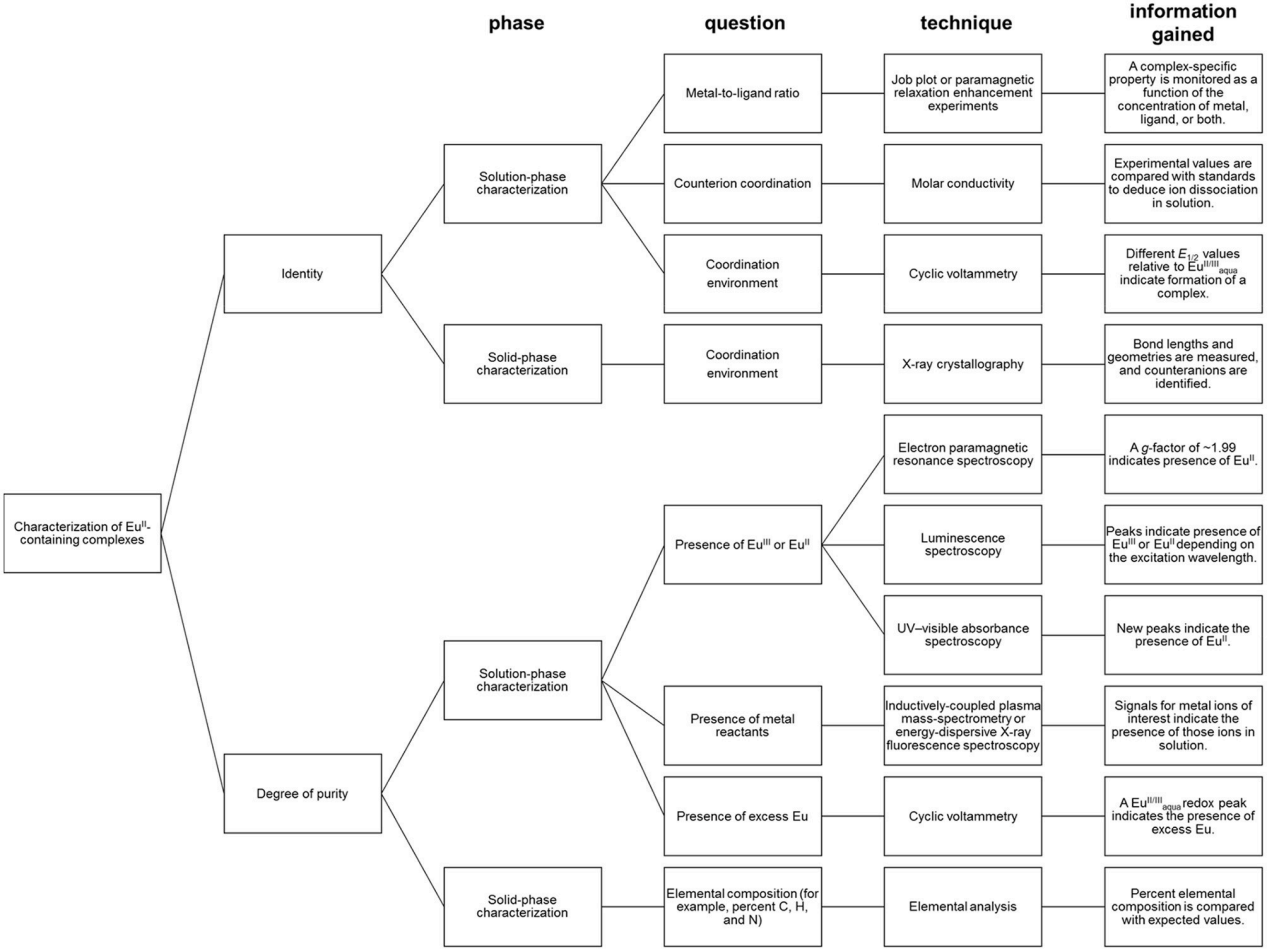
Diagram for the characterization of Eu^II^-containing complexes.

Depending the route chosen to form Eu^II^-containing complexes, different techniques for the characterization of identity and purity of Eu^II^-containing complexes must be used (Figure 5). Assessment of identity of Eu^II^-containing complexes includes evidence for the oxidation state of Eu, coordination environment, and metal-to-ligand ratio. Assessment of the purity of Eu^II^-containing complexes includes the detection of Eu^II^ or Eu^III^, ligand, reactants, or byproducts.

When Eu^II^-containing complexes are generated *in situ* via cyclic voltammetry, purity with respect to excess ${\text{Eu}}_{\text{aqua}}^{\text{II}}$ can be assessed by observation of a peak for ${\text{Eu}}_{\text{aqua}}^{\text{II}/\text{III}}$. The minimum detectable concentration of europium by cyclic voltammetry, and hence the boundary of usefulness for this technique, is influenced by multiple experimental parameters including concentration of supporting electrolyte, the identity of the buffer and solvent, and choice of reference electrode (Bard and Faulkner, 2000). With these parameters in mind, minimum detectable concentrations can be determined experimentally (Harris, 2003). Unlike Eu^II/III^, if a ligand is not redox-active, cyclic voltammetry does not provide evidence for the presence of uncomplexed ligand. Therefore, the usefulness of cyclic voltammetry for detection of excess ligand is situationally dependent.

Regarding the identity of the complex that is formed during the course of cyclic voltammetry, formation of a Eu-containing complex can be validated by comparing the standard electrode potential of the new complex with the standard electrode potential of a sample of the Eu-containing complex (Tables 1, 2). For example, Eu^II^-containing complexes of **8**, **20**, and **21** were synthesized and found to produce the same *E*_1/2_ whether starting with a mixture of Eu^III^ and ligand or an already-synthesized Eu^II^-containing complex (Yee et al., 1980, 1983). However, the standard electrode potential is influenced by the same experimental parameters that are listed for consideration of purity using cyclic voltammetry; therefore, care must be taken to note experimental parameters when comparing standard electrode potentials.

**Table 1:** Midpoint potentials of Eu^II/III^-containing complexes more negative than aqua ions.

**Ligand**	***E*_1/2_ (V)^[a]^**	***E*_1/2_ (V)**	**Reference electrode**	**pH**	**References**
**5**	−1.417	−1.373	saturated calomel	4	Botta et al., 2003
**4**	−1.386	−1.342	saturated calomel	4	Botta et al., 2003
**23**	−1.375	−1.331	saturated calomel	4	Botta et al., 2003
**4**	−1.356	−1.356	Ag/AgCl	6.5	Gál et al., 2013
**2**	−1.356	−1.312	saturated calomel	4	Botta et al., 2003
**4**	−1.35	−1.35	Ag/AgCl	nr^[b]^	Burai et al., 2000
**38**	−1.331	−1.331	Ag/AgCl	6.5	Gál et al., 2013
**37**	−1.291	−1.291	Ag/AgCl	6.5	Gál et al., 2013
**31**	−1.241	−1.241	Ag/AgCl	6.5	Gál et al., 2013
**39**	−1.18	−1.18	Ag/AgCl	7	Vanek et al., 2016
**22**	−1.164	−1.120	saturated calomel	4	Botta et al., 2003
**3**	−1.153	−1.109	saturated calomel	4	Botta et al., 2003
**31**	−1.135	−1.135	Ag/AgCl	nr^[b]^	Burai et al., 2003
**1**	−1.125	−1.081	Saturated calomel	4	Botta et al., 2003
**32**	−1.1105	−1.1105	Ag/AgCl	7	Burnett et al., 2017
**33**	−1.0680	−1.0680	Ag/AgCl	7	Burnett et al., 2017
**36**	−1.052	−1.052	Ag/AgCl	6.5	Gál et al., 2013
**25**	−0.996	−0.996	Ag/AgCl	nr^[b]^	Burai et al., 2003
**34**	−0.9710	−0.9710	Ag/AgCl	7	Burnett et al., 2017
**6**	−0.92	−0.92	Ag/AgCl	nr^[b]^	Burai et al., 2000
**34**	−0.903	−0.903	Ag/AgCl	7	Ekanger et al., 2016a
**34**	−0.879	−0.879	Ag/AgCl	4	Ekanger et al., 2016a
**7**	−0.82	−0.82	Ag/AgCl	nr^[b]^	Burai et al., 2000
**35**	−0.817	−0.817	Ag/AgCl	6.5	Gál et al., 2013
**28**	−0.753	−0.753	Ag/AgCl	7	Regueiro-Figueroa et al., 2015
**26**	−0.727	−0.727	Ag/AgCl	na^[c]^	Basal et al., 2017a
**27**	−0.720	−0.720	Ag/AgCl	na^[c]^	Basal et al., 2017a

[a] *converted to V vs. Ag/AgCl by subtracting 0.044 V from the saturated calomel values (Bard and Faulkner, 2000)*;

[b] *nr, not reported*;

[c] *na, not applicable, solvent is dimethylformamide*.

**Table 2:** Midpoint potentials of Eu^II/III^-containing aqua complexes and complexes more positive than aqua ions.

**Ligand**	***E*_1/2_ (V)^[a]^**	***E*_1/2_ (V)**	**Reference electrode**	**pH**	**References**
aqua	−0.67	−0.67	Ag/AgCl	6.5	Gál et al., 2013
aqua	−0.668	−0.624	saturated calomel	4	Botta et al., 2003
aqua	−0.664	−0.620	saturated calomel	3	Yee et al., 1979
aqua	−0.648	−0.648	Ag/AgCl	na^[d]^	Basal et al., 2017a
aqua	−0.6365	−0.6365	Ag/AgCl	7	Burnett et al., 2017
aqua	−0.63	−0.63	Ag/AgCl	nr^[e]^	Burai et al., 2000
**30**	−0.61	−0.61	Ag/AgCl	7	Regueiro-Figueroa et al., 2015
aqua	−0.585	−0.585	Ag/AgCl	nr^[e]^	Burai et al., 2003
aqua	−0.554	−0.554	Ag/AgCl	2.08	Anderson and Macero, 1963
aqua	−0.549	−0.7645^[b]^	Fc/Fc^+^^[c]^	7.5	Gamage et al., 2010
**20**	−0.479	−0.435	saturated calomel	2–7	Yee et al., 1980
**29**	−0.453	−0.453	Ag/AgCl	7	Regueiro-Figueroa et al., 2015
**21**	−0.41	−0.37	saturated calomel	nr^[e]^	Yee et al., 1980
**8**	−0.259	−0.215	saturated calomel	nr^[e]^	Yee et al., 1980
**8**	−0.141	−0.3669^[b]^	Fc/Fc^+^^[c]^	7.5	Gamage et al., 2010
[P_5_W_30_O_110_]^15−^	0.11	0.11	Ag/AgCl	0	Antonio and Soderholm, 1996
**14**	−0.100	−0.2996^[b]^	Fc/Fc^+^^[c]^	7.5	Gamage et al., 2010
**11**	−0.068	−0.2769^[b]^	Fc/Fc^+^^[c]^	7.5	Gamage et al., 2010
**10**	0.016	−0.2324^[b]^	Fc/Fc^+^^[c]^	7.5	Gamage et al., 2010
**13**	0.083	−0.2123^[b]^	Fc/Fc^+^^[c]^	7.5	Gamage et al., 2010
**16**	0.13	0.13	Ag/AgCl	9.7	Kuda-Wedagedara et al., 2015

[a] *converted to V vs. Ag/AgCl by subtracting 0.044 V from the saturated calomel values (Bard and Faulkner, 2000) or by adding |E_pa(Ag/AgCl)_-E_pa(ferrocene/ferrocenium)_| to the reported E_1/2_ values vs. ferrocene/ferrocenium (Gamage et al., 2010)*;

[b] *average of the anodic and cathodic peak potentials*;

[c] *Fc/Fc^+^, ferrocene/ferrocenium*;

[d] *na, not applicable, solvent is dimethylformamide*;

[e] *nr, not reported*.

In the case where Eu^III^-containing complexes are reduced chemically with zinc, such as 2Eu**22**Cl_3_ + Zn^0^ → 2Eu**22**Cl_2_ + ZnCl_2_, a combination of spectroscopic techniques can be used to provide evidence of the oxidation state and degree of purity. For evidence that Zn^II^ was removed from solution post-reduction, the concentration of Zn^II^ (down to parts-per-billion levels) can be monitored with inductively coupled plasma–mass spectrometry (Ekanger et al., 2016a). For evidence of the reduction of Eu^III^, a lack of overlap of the excitation bands of Eu^II^- and Eu^III^-containing species enables monitoring of the presence of Eu^III^ (down to micromolar levels) by luminescence spectroscopy when excitation is performed with a Eu^III^-specific wavelength (Ekanger et al., 2016a; Basal et al., 2017a). Evidence for the generation of Eu^II^ is obtained using electron paramagnetic resonance (EPR) spectroscopy. In its ground state, Eu^III^ has no net magnetic moment (Cullity and Graham, 2009) despite having six unpaired electrons. The magnetic moment (μ_eff_) of lanthanides is calculated using the total angular momentum (*J*), unlike the magnetic moments of 3d^*n*^ transition metals that take into account the number of unpaired electrons (Cotton, 2006; Layfield et al., 2015). This difference is due to the quenching of orbital angular momentum by ligands for 3d orbitals but not for the shielded 4f orbitals. Therefore, the Eu^III^ ground state would not be expected to be observed in EPR spectroscopy (Abragam and Bleaney, 1970). However, Eu^II^ is paramagnetic and characterized by a signal in EPR spectroscopy with a *g* factor of ~1.99 (Abragam and Bleaney, 1970; Caravan et al., 1999). Also, Eu^II^-containing complexes can be colored yellow or orange and give rise to broad and relatively intense UV–visible absorptions and emissions that are distinct from the corresponding Eu^III^-containing species (Burai et al., 2003; Kuda-Wedagedara et al., 2015; Ekanger et al., 2016a). For example, a combination of spectroscopic techniques were used to monitor the formation of Eu^II^-containing complexes Eu**22**, Eu**26**, and Eu**34** (Ekanger et al., 2016a; Basal et al., 2017a).

In the case where the formation of a complex was achieved by mixing EuCl_2_ with ligands, evidence of 1:1 complex formation in solution was determined by measuring the change in relaxivity as a function of Eu^II^-to-ligand ratio, a technique known as proton relaxation enhancement (Lauffer, 1987; Lenora et al., 2017). Another solution-phase technique to monitor complex formation is a Job plot where both ligand and metal ratios are varied, and a unique property of the complex, such as a complex-specific emission, is monitored (Renny et al., 2013; Kuda-Wedagedara et al., 2015). The choice of spectral feature to monitor in a Job plot is complex-dependent. Typically, Eu^II^-based emission is largely quenched in aqueous media due to the abundance of OH oscillators (Jiang et al., 1998); therefore, luminescence spectroscopy is not suitable to characterize the formation of every Eu^II^-containing complex. Other features to monitor as a function of metal-to-ligand ratio include complex-specific absorbance peaks, relaxivity, or cyclic voltammetry peaks.

In the case where single crystals of a Eu^II^-containing complex are obtained, X-ray diffraction combined with elemental analysis provides information regarding identity and purity. X-ray diffraction provides information about the oxidation state and coordination environment of the Eu^II^ ion in the solid state, including bond distances and number and identity of counter ions (Zucchi et al., 2010; Ekanger et al., 2015; Kuda-Wedagedara et al., 2015; Jin et al., 2016; Basal et al., 2017a; Lenora et al., 2017). Elemental analysis provides information about elemental composition as an indication of purity. However, it is important to note that for molecular-imaging applications, solution-phase characterization is often more important than solid-phase analysis of solids because solid-state properties do not necessarily reflect solution-phase behavior or coordination environment. For example, Eu^II^-containing complexes Eu**10**, Eu**11**, and Eu**16**, crystallize with one chloride counteranion bound to Eu^II^. In the case of Eu**16**, solution-phase characterization suggests that the counteranion remains coordinated to Eu^II^ in solution (Kuda-Wedagedara et al., 2015); however, for Eu**10** and Eu**11**, molar-conductivity data suggests that counteranions dissociate in solution (Ekanger et al., 2015; Lenora et al., 2017).

## Handling Eu^II^-containing samples to prevent oxidation

**Figure 6:**
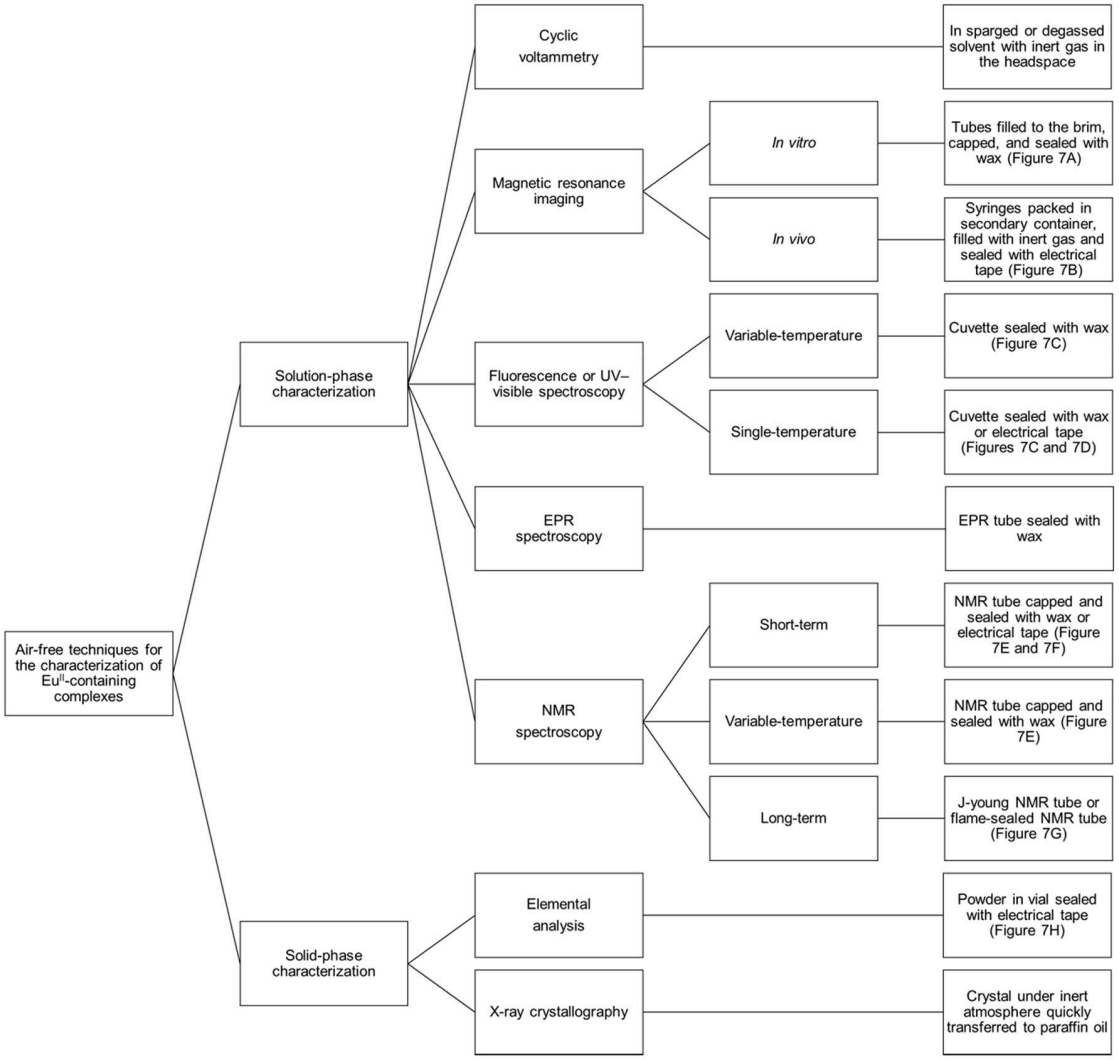
Diagram describing techniques that can be used to analyze Eu^II^-containing complexes with air-free conditions.

**Figure 7:**
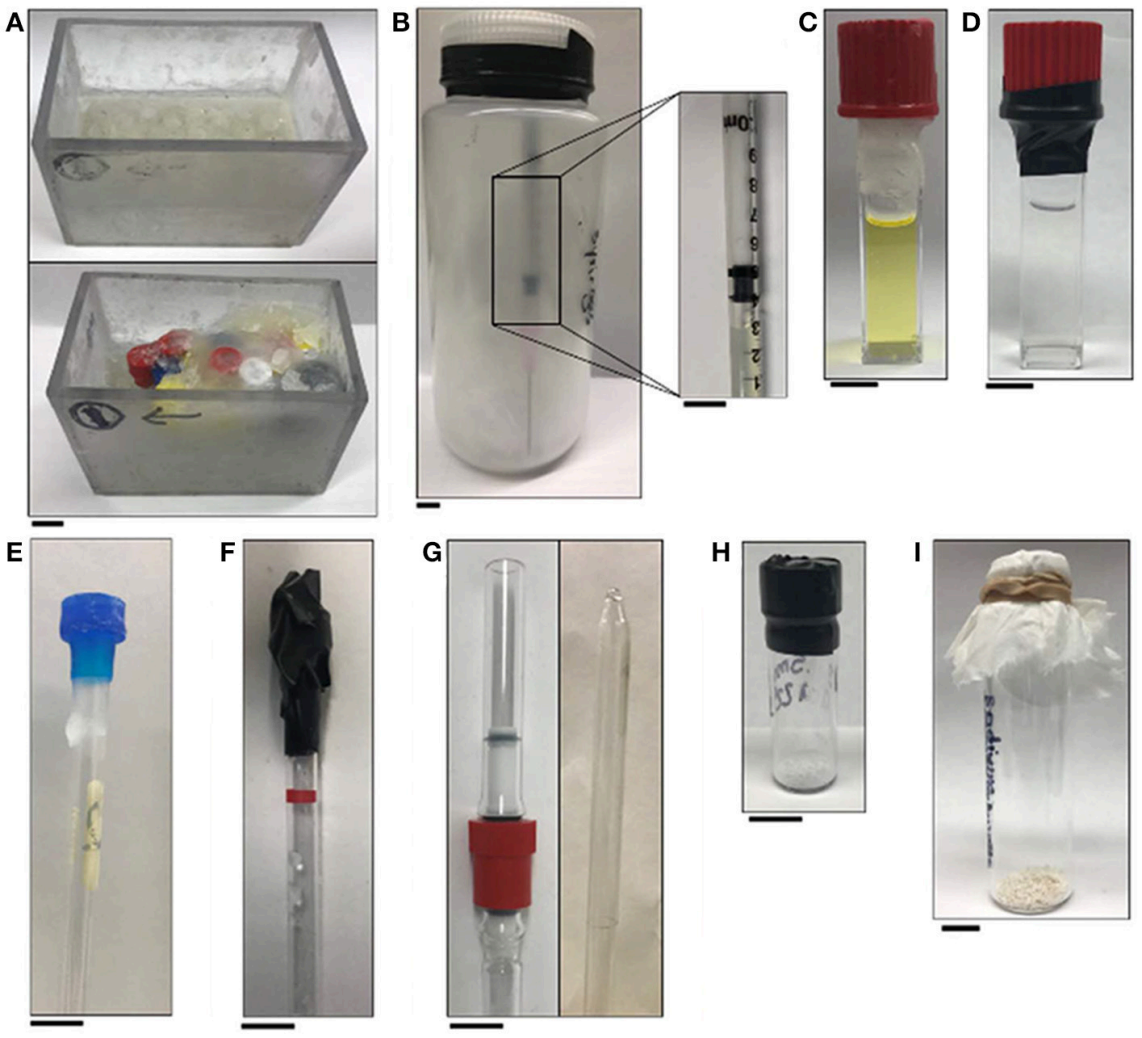
Pictures of apparatuses included in the handling section of this manuscript: **(A)** empty tube holder (top) and tube holder with tubes that are covered in wax (bottom); **(B)** jar sealed with electrical tape that contains glass wool and syringes of Eu^II^-containing complexes packed under an inert atmosphere and (inset) a plastic 1 mL syringe with a rubber tip on the plunger that contains a solution of a Eu^II^-containing complex; **(C)** wax-sealed cuvette; **(D)** electrical-tape-sealed cuvette; **(E)** wax-sealed NMR tube; **(F)** electrical-tape-sealed NMR tube; **(G)** J-young NMR tube (left) and flame-sealed NMR tube (right); **(H)** electrical-tape-sealed vial containing a solid sample; and **(I)** glass vial that contains solid sample covered with a tissue that is secured with a rubber band. All scale bars represent 1 cm.

Preventing Eu^II^-containing complexes from oxidizing over the course of analyses is critical for the collection of accurate data: Eu^III^ and Eu^II^ have different properties, and misinterpretation of experimental results can occur if care is not taken to prevent unintentional oxidation of Eu^II^. Rigorous techniques must be used in the synthesis and handling of Eu^II^-containing complexes. This section describes apparatuses and techniques that were successfully used to study Eu^II^-containing complexes (Figures 6, 7).

Cyclic voltammetry or bulk electrolysis performed either inside or outside of a glovebox should use solvents that have been degassed (under reduced pressure, for example, on a Schlenck line) or well-sparged (≥5 min of vigorous bubbling with an inert gas for volumes of ~3 mL in a capped vessel that contains a vent needle). To ensure that there is no detectable dissolved oxygen in solution, cyclic voltammetry of degassed solvents should not show peaks for O_2_ (Green, 2018). If outside of a glovebox, the inert-gas source should be retracted to the head-space of the vessel once sparging is complete prior to cyclic voltammetry or bulk electrolysis. Inside the glovebox, there is no need for an inert gas line in the headspace because the atmosphere is O_2_-free. If outside of a glovebox, transfer of solutions of Eu^II^ from bulk electrolysis for crystal growth or other analyses must be performed using air-free techniques (Shriver and Drezdzon, 1986).

For other routes to synthesize and handle Eu^II^-containing complexes in aqueous media, air-free handling is achieved using Shlenck techniques, a wet (water allowed but no molecular oxygen) glovebox, or a combination of both. During the synthesis of Eu^II^-containing complexes in a glovebox, the glovebox atmosphere must not be contaminated with oxygen. Either a commercial oxygen sensor or chemical indicator can be used to monitor the atmosphere. One suitable chemical indicator for this purpose is dicyclopentadienyltitanium(IV) dichloride (Ti^IV^Cp_2_Cl_2_) (Burgmayer, 1998). When the titanium metallocene is dissolved in acetonitrile in the presence of zinc metal, a deep blue solution is obtained. An aliquot of this solution is filtered through celite or a hydrophilic filter and diluted with acetonitrile to yield a diffuse, blue solution caused by the presence of Ti^III^Cp_2_(NCCH_3_)_2_. If the solution remains blue upon evaporation, Ti^III^Cp_2_(NCCH_3_)_2_ is unoxidized, indicating that the atmosphere is good (<5 ppm O_2_) (Shriver and Drezdzon, 1986). A color change to green is caused by formation of a dimeric species or some oxidation of Ti^III^ to Ti^IV^. A color change to yellow is indicative of near-complete oxidation to Ti^IV^Cp_2_(NCCH_3_)_2_. Either green or yellow suggests a bad atmosphere with respect to O_2_, and steps to address the quality of the atmosphere should be taken prior to working with Eu^II^. If the indicator persists as green or yellow after refreshing the glovebox atmosphere by purging the glovebox with inert gas, then the oxygen-removing catalyst should be replaced or regenerated. Ideally, the atmosphere of a wet glovebox should be checked at least daily, and the atmosphere should be purged with inert gas before and after each use. All liquids to be used in a wet glovebox should be rigorously degassed before transport into the glovebox. Solids to be brought into a glovebox can be placed in an open vial and brought into the antechamber if the solids do not sublime at the temperature and pressure of the glovebox antechamber. To prevent loss of solid from bumping or the vial accidentally tipping, the top of the vial can be covered with tissue that is secured with a rubber band (Figure 7I). If solids sublime at the temperature and pressure of the antechamber, then the solids should be placed under an inert atmosphere in a sealed container prior to being brought into the glovebox.

Solution-phase characterization of Eu^II^-containing samples, including NMR spectroscopy, MRI, and fluorescence or UV–visible absorbance spectroscopy, requires that samples be sealed to prevent oxygen contamination that would interfere with the integrity of the results. For NMR spectroscopy, J-young NMR tubes with Teflon seals or flame-sealed NMR tubes are appropriate for long term studies (Figure 7G). Alternatively, NMR tubes capped with a plastic cap and sealed with paraffin wax or electrical tape suffice for studies that last a few hours (Figures 7E,F and Video S1). For samples that must be shipped, samples can be loaded into NMR tubes that are subsequently flame-sealed (Lenora et al., 2017). For MRI, tubes (for example, glass vials that have a 400 μL capacity) can be filled to the brim with solution (to avoid bubbles), capped, dipped in wax, and loaded into an apparatus that is then covered in paraffin wax (Figure 7A) (Garcia and Allen, 2012b; Garcia et al., 2012; Basal et al., 2017a,b). For *in-vivo* injections, syringes with rubber-tip plungers can be loaded with sample, packaged in a bottle that is under an atmosphere of N_2_ or Ar, and sealed with electrical tape (Figure 7B; Basal et al., 2017a). Packed this way, the integrity of samples is sufficient for shipping with glass wool included in the bottle to minimize vibrations during shipping. For samples in cuvettes (for example, samples for emission or absorbance spectroscopy that must be removed from the glovebox), quartz cuvettes with Teflon caps can be sealed with paraffin wax (Figure 7C) or electrical tape (Figure 7D) (Kuda-Wedagedara et al., 2015; Ekanger et al., 2016a; Jin et al., 2016; Basal et al., 2017a). For samples that will undergo temperature changes (heating or cooling), we have observed that paraffin wax is more reliable than electrical tape.

To ensure that a technique or apparatus successfully seals Eu^II^ from air over the course of an experiment, the relaxivity, UV–visible absorption, or luminescence spectra of the Eu^II^-containing complex can be measured immediately after sample preparation and after the analyses are complete, if the analyses are nondestructive. Another way to assess the air-free environment and handling of a Eu^II^-containing sample is to measure a spectral feature of the Eu^II^-containing complex as a function of time at different concentrations of europium. Time-dependent measurements can reveal the presence of oxidizing impurities (Burai et al., 2002). For example, the oxidation half-life (*t*_1/2_), which is the time at which half of the complex has oxidized, was measured for Eu**6** by monitoring the intensity of a complex-specific UV–visible absorbance peak as a function of time (Burai et al., 2002). For a 5 mM solution of Eu**6**, the *t*_1/2_ was found to be 10 days. However, *t*_1/2_ increased as a function of concentration of europium (>1 month for Eu**6** at 100 mM), suggesting that the *t*_1/2_ reported at 5 mM was influenced by the presence of oxidizing impurities such as O_2_. Oxygen can be avoided using the techniques described in this section.

If a Eu^II^-containing sample cannot be monitored to check the effectiveness of an air-free technique, then another way to assess if an apparatus is sealed from air is to monitor the color change of a solution of Ti^III^Cp_2_Cl_2_ sealed in parallel to the sample to be measured (Burgmayer, 1998). The use of Ti^III^Cp_2_Cl_2_ as an indicator provides information regarding the technique used to seal the solution from air. However, a limitation of this method is that it does not provide direct information about the Eu^II^-containing complex being analyzed.

## Conclusions and outlook

The unique and tuneable properties of Eu^II^ make Eu^II^-containing complexes promising molecular imaging agents. The synthesis, characterization, and handling of Eu^II^ require care with respect to the use of air-free techniques and characterization of oxidation states because Eu^II^ and Eu^III^ have different molecular imaging properties that confound results if the ions are inadvertently comingled within a sample. We expect that the techniques described in this review will guide the future synthesis, characterization, and handling of Eu^II^-containing complexes for molecular imaging.

## Author contributions

LB and MA: Contributed to the manuscript and approved the final version.

### Conflict of interest statement

The authors declare that the research was conducted in the absence of any commercial or financial relationships that could be construed as a potential conflict of interest.

## Supplementary material

Video S1 Video demonstration of the sealing of an NMR sample with paraffin wax.
